# The genome sequence of a cranefly, *Tipula *( *Savtshenkia*) *confusa *van der Wulp, 1883

**DOI:** 10.12688/wellcomeopenres.22470.1

**Published:** 2024-06-24

**Authors:** Duncan Sivell, Natural History Museum Genome Acquisition Lab, Darwin Tree of Life Barcoding collective, Wellcome Sanger Institute Tree of Life Management, Samples and Laboratory team, Wellcome Sanger Institute Scientific Operations: Sequencing Operations, Wellcome Sanger Institute Tree of Life Core Informatics team, Tree of Life Core Informatics collective, Darwin Tree of Life Consortium

**Affiliations:** 1 Natural History Museum, London, England, UK

**Keywords:** Tipula confusa, cranefly, genome sequence, chromosomal, Diptera

## Abstract

We present a genome assembly from an individual male *Tipula confusa* (cranefly; Arthropoda; Insecta; Diptera; Tipulidae). The genome sequence is 728.1 megabases in span. Most of the assembly is scaffolded into 5 chromosomal pseudomolecules, including the X and Y sex chromosomes. The mitochondrial genome has also been assembled and is 16.94 kilobases in length.

## Species taxonomy

Eukaryota; Opisthokonta; Metazoa; Eumetazoa; Bilateria; Protostomia; Ecdysozoa; Panarthropoda; Arthropoda; Mandibulata; Pancrustacea; Hexapoda; Insecta; Dicondylia; Pterygota; Neoptera; Endopterygota; Diptera; Nematocera; Tipulomorpha; Tipuloidea; Tipulidae; Tipulinae; *Tipula; Savtshenkia*; *Tipula confusa* van der Wulp, 1883 (NCBI:txid2881124).

## Background

*Tipula confusa* is a western Palaearctic species with an Atlantic distribution ranging from Morocco across Iberia and northern Europe into Scandinavia and western Russia ( Dufour, 1986; Jakovlev *et al.*, 2014; Kettani *et al.*, 2022). *Tipula confusa* is found throughout Britain from the Isles of Scilly to Shetland ( Stubbs, 1992) and is common in a variety of habitats, including urban areas ( Boardman, 2016; Stubbs, 2021). The larvae live in moss ( Brindle, 1960; Dufour, 1986; Stubbs, 1992) and have also been found under the bark of dead wood ( Alexander, 2002).

*Tipula confusa* is a medium-sized cranefly (wing length 10–16 mm) with a grey head and thorax, a dull orange to grey abdomen and mottled grey wings. This general appearance is shared by several craneflies in the *Savtshenkia* subgenus. In *T. confusa* the pale mark in the lower basal cell of the wing is usually V-shaped, but this character can be variable. Males have a deep and wide horseshoe-shaped indentation in sternite 8 that is distinctive. The identification of female *T. confusa* may require more care. Recent keys are available in Boardman (2016) and Stubbs (2021). Larvae and pupae can be identified using Brindle (1960).

This is an autumn species that is on the wing between June and November ( Boardman, 2016). *Tipula confusa* can be abundant on moorland in late August and early September and on lower ground can be very common in woodlands and urban areas in September and October ( Stubbs, 2021). This species has been recorded in May, but these are rare instances and do not indicate a wider spring emergence.

*Tipula confusa* is thought to tolerate drier conditions than most craneflies ( Brindle, 1960; Stubbs, 1992), but droughts during the 1990s appear to have weakened populations in southern England ( Stubbs, 2021). Changes in the abundance of this common and widespread species may be symptomatic of the effects of climate change at a regional level.

In older literature this species is referred to as *Tipula* ( *Savtshenkia*) *marmorata* Meigen, 1818 ( Brindle, 1960; Coe, 1950; Stubbs, 1972). *Tipula confusa* has also been referred to by the common names “mottled Autumn Tipula” ( Stubbs, 1972) and “U-tailed mottle” ( Stubbs, 2021).

The genome of *Tipula confusa* was sequenced as part of the Darwin Tree of Life Project, a collaborative effort to sequence all named eukaryotic species in the Atlantic Archipelago of Britain and Ireland. Here we present a chromosomally complete genome sequence based on one male specimen (NHMUK014561543) collected on 27/09/2021 from a private urban garden in Luton (51.88, –0.38). The specimen was identified by DS using Boardman (2016) and Stubbs (2021). This genome note will aid research on the phylogeny, taxonomy, biology and ecology of the species.

## Genome sequence report

**Figure 1.**
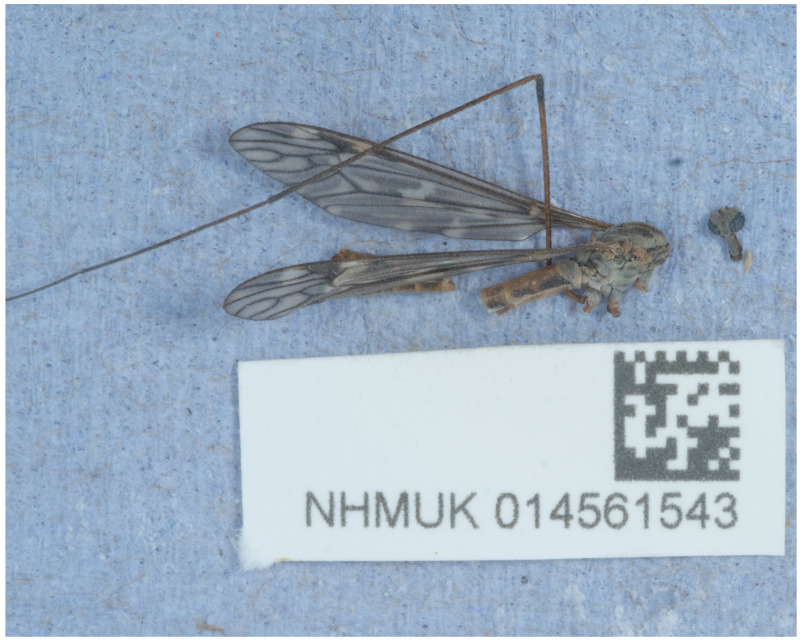
Photograph of the *Tipula confusa* (idTipConf1) specimen used for genome sequencing.

The genome was sequenced from adult male *Tipula confusa* ( Figure 1) collected from Luton, England, UK (51.89, –0.38). A total of 32-fold coverage in Pacific Biosciences single-molecule HiFi long reads was generated. Primary assembly contigs were scaffolded with chromosome conformation Hi-C data. Manual assembly curation corrected 153 missing joins or mis-joins and removed 22 haplotypic duplications, reducing the assembly length by 0.68% and the scaffold number by 13.30%, and increasing the scaffold N50 by 2.54%.

**Figure 2.**
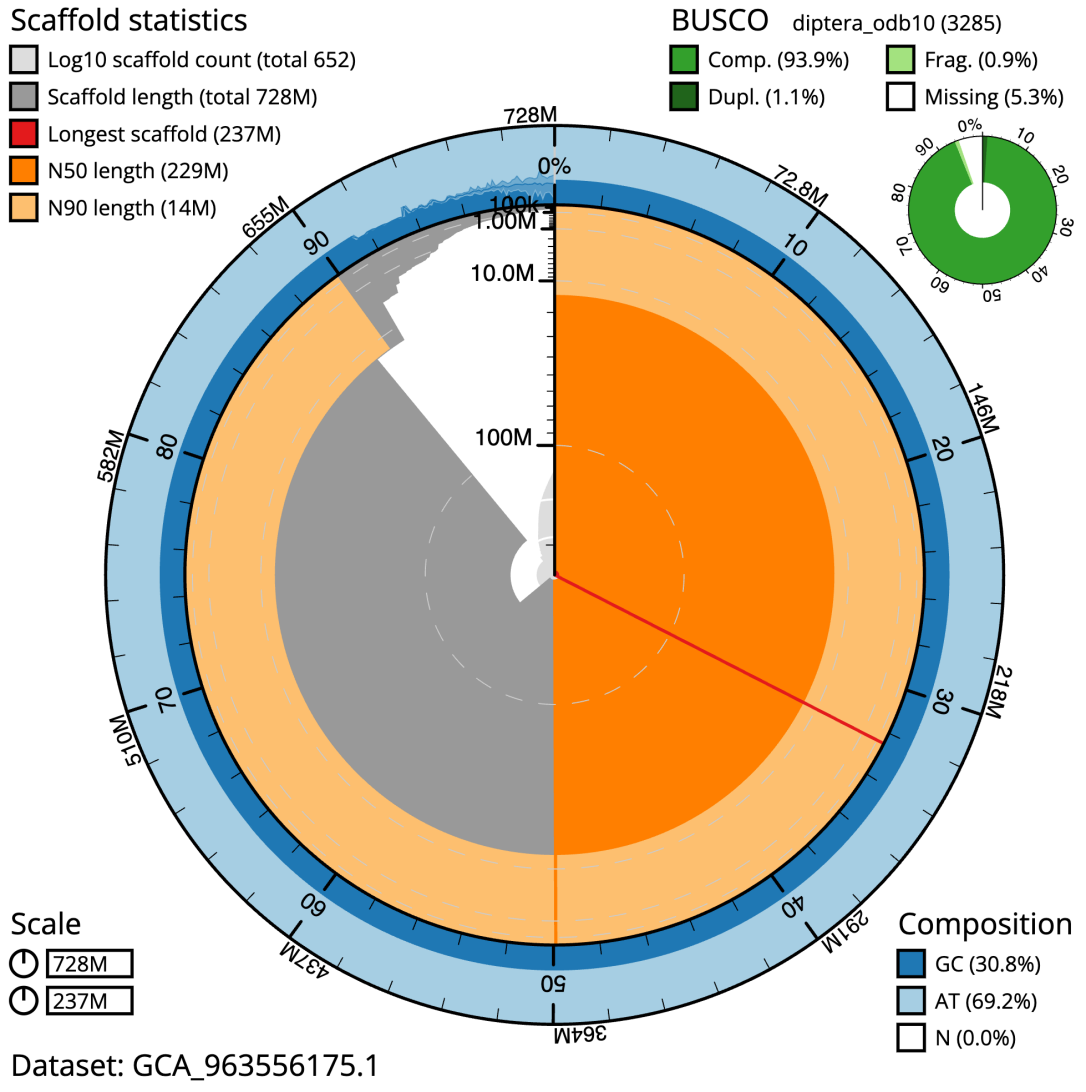
Genome assembly of *Tipula confusa*, idTipConf1.1: metrics. The BlobToolKit snail plot shows N50 metrics and BUSCO gene completeness. The main plot is divided into 1,000 size-ordered bins around the circumference with each bin representing 0.1% of the 728,119,463 bp assembly. The distribution of scaffold lengths is shown in dark grey with the plot radius scaled to the longest scaffold present in the assembly (236,655,262 bp, shown in red). Orange and pale-orange arcs show the N50 and N90 scaffold lengths (228,831,665 and 14,021,489 bp), respectively. The pale grey spiral shows the cumulative scaffold count on a log scale with white scale lines showing successive orders of magnitude. The blue and pale-blue area around the outside of the plot shows the distribution of GC, AT and N percentages in the same bins as the inner plot. A summary of complete, fragmented, duplicated and missing BUSCO genes in the diptera_odb10 set is shown in the top right. An interactive version of this figure is available at https://blobtoolkit.genomehubs.org/view/Tipula_confusa/dataset/GCA_963556175.1/snail.

**Figure 3.**
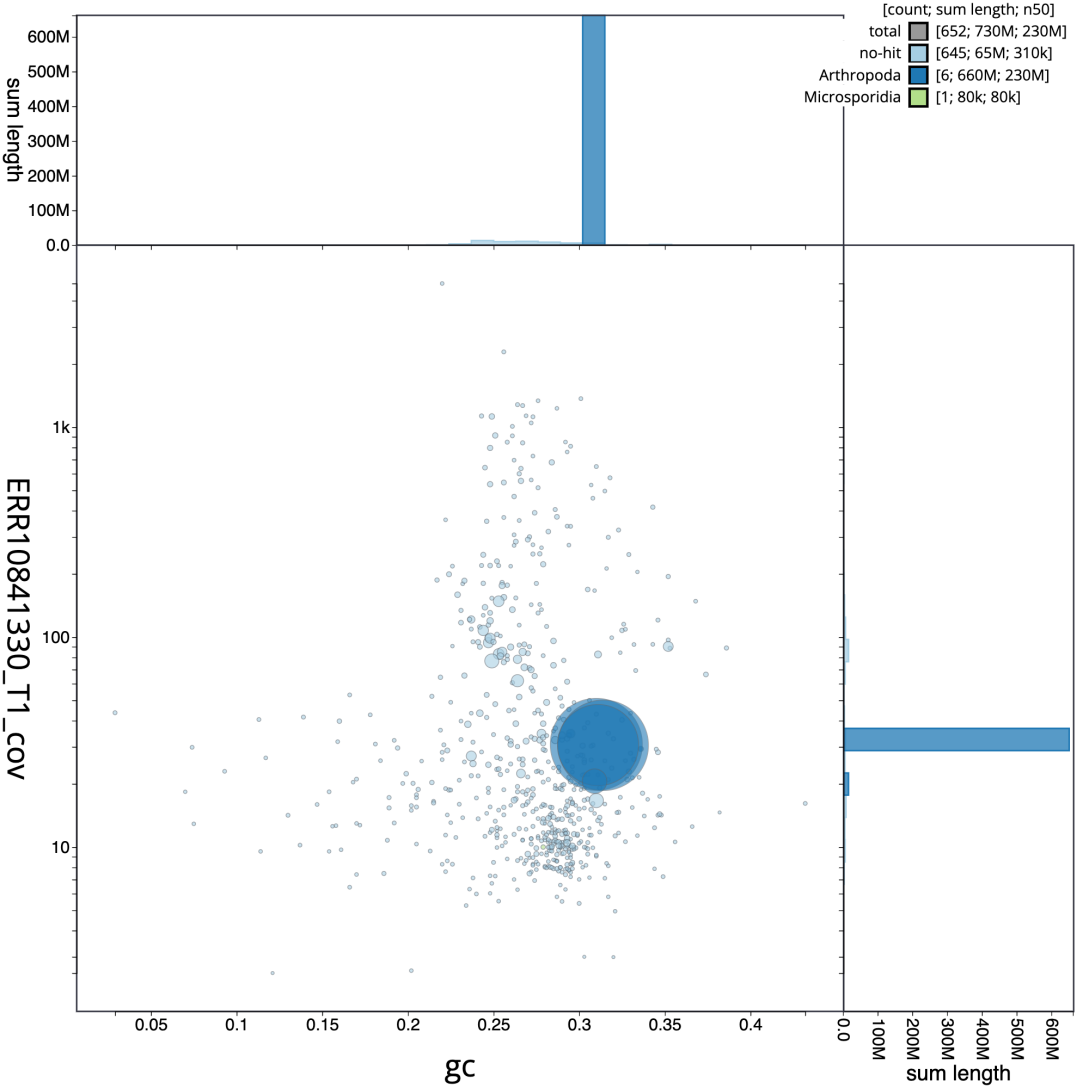
Genome assembly of *Tipula confusa*, idTipConf1.1: BlobToolKit GC-coverage plot. Sequences are coloured by phylum. Circles are sized in proportion to sequence length. Histograms show the distribution of sequence length sum along each axis. An interactive version of this figure is available at https://blobtoolkit.genomehubs.org/view/Tipula_confusa/dataset/GCA_963556175.1/blob.

**Figure 4.**
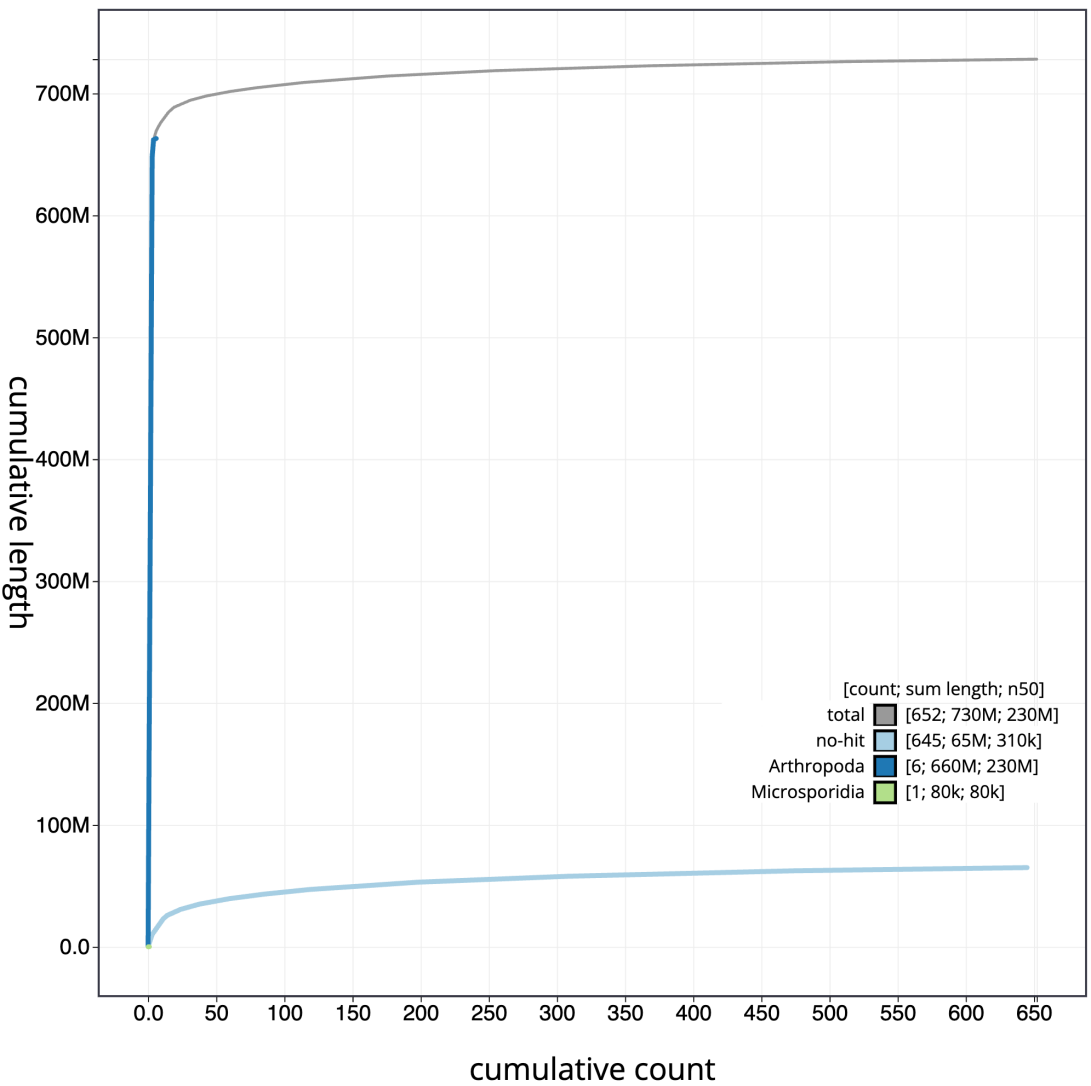
Genome assembly of *Tipula confusa* idTipConf1.1: BlobToolKit cumulative sequence plot. The grey line shows cumulative length for all sequences. Coloured lines show cumulative lengths of sequences assigned to each phylum using the buscogenes taxrule. An interactive version of this figure is available at https://blobtoolkit.genomehubs.org/view/Tipula_confusa/dataset/GCA_963556175.1/cumulative.

**Figure 5.**
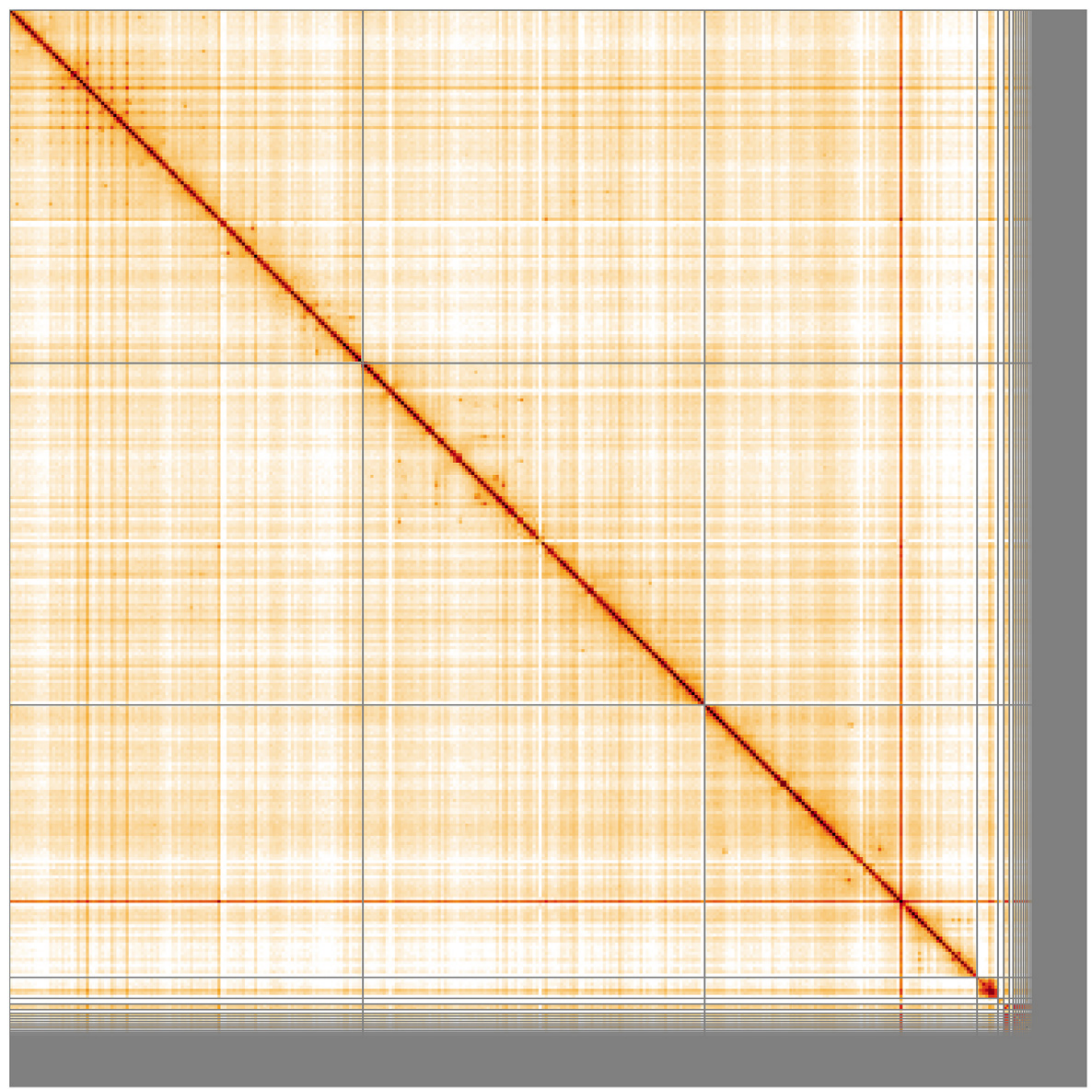
Genome assembly of *Tipula confusa* idTipConf1.1: Hi-C contact map of the idTipConf1.1 assembly, visualised using HiGlass. Chromosomes are shown in order of size from left to right and top to bottom. An interactive version of this figure may be viewed at https://genome-note-higlass.tol.sanger.ac.uk/l/?d=Vk-QIJ-ySei2cTOFY6J7-w.

The final assembly has a total length of 728.1 Mb in 651 sequence scaffolds with a scaffold N50 of 228.8 Mb ( Table 1). The snail plot in Figure 2 provides a summary of the assembly statistics, while the distribution of assembly scaffolds on GC proportion and coverage is shown in Figure 3. The cumulative assembly plot in Figure 4 shows curves for subsets of scaffolds assigned to different phyla. Most (91.52%) of the assembly sequence was assigned to 5 chromosomal-level scaffolds, representing 3 autosomes and the X and Y sex chromosomes. Chromosome-scale scaffolds confirmed by the Hi-C data are named in order of size ( Figure 5; Table 2). While not fully phased, the assembly deposited is of one haplotype. Contigs corresponding to the second haplotype have also been deposited. The mitochondrial genome was also assembled and can be found as a contig within the multifasta file of the genome submission.

**Table 1.** Genome data for *Tipula confusa*, idTipConf1.1.

Project accession data		
Assembly identifier	idTipConf1.1	
Species	*Tipula confusa*	
Specimen	idTipConf1	
NCBI taxonomy ID	2881124	
BioProject	PRJEB59392	
BioSample ID	PacBio sequencing: SAMEA111458222 Hi-C scaffolding: SAMEA111457937	
Isolate information	idTipConf1: thorax (genome sequencing), head (Hi-C sequencing)	
Assembly metrics *		*Benchmark*
Consensus quality (QV)	54.0	*≥ 50*
*k*-mer completeness	99.99%	*≥ 95%*
BUSCO **	C:93.6%[S:92.6%,D:1.0%],F:1.0%, M:5.4%,n:3,285	*C ≥ 95%*
Percentage of assembly mapped to chromosomes	91.52%	*≥ 95%*
Sex chromosomes	XY	*localised homologous pairs*
Organelles	Mitochondrial genome: 16.94 kb	*complete single alleles*
Raw data accessions		
PacificBiosciences Sequel IIe	ERR10841330	
Hi-C Illumina	ERR10851536	
Genome assembly		
Assembly accession	GCA_963556175.1	
*Accession of alternate haplotype*	GCA_963556025.1	
Span (Mb)	728.1	
Number of contigs	1944	
Contig N50 length (Mb)	1.2	
Number of scaffolds	651	
Scaffold N50 length (Mb)	228.8	
Longest scaffold (Mb)	236.66	

* Assembly metric benchmarks are adapted from column VGP-2020 of “Table 1: Proposed standards and metrics for defining genome assembly quality” from Rhie *et al.* (2021). ** BUSCO scores based on the diptera_odb10 BUSCO set using version 5.4.3. C = complete [S = single copy, D = duplicated], F = fragmented, M = missing, n = number of orthologues in comparison. A full set of BUSCO scores is available at https://blobtoolkit.genomehubs.org/view/Tipula_confusa/dataset/GCA_963556175.1/busco.

**Table 2.** Chromosomal pseudomolecules in the genome assembly of *Tipula confusa*, idTipConf1.

INSDC accession	Chromosome	Length (Mb)	GC%
OY744475.1	1	236.66	31.0
OY744476.1	2	228.83	31.5
OY744477.1	3	182.39	31.0
OY744478.1	X	14.02	31.0
OY744479.1	Y	3.84	31.0
OY744480.1	MT	0.02	22.0

**Table 3.** Software tools: versions and sources.

Software tool	Version	Source
BEDTools	2.30.0	https://github.com/arq5x/bedtools2
Blast	2.14.0	ftp://ftp.ncbi.nlm.nih.gov/blast/executables/blast+/
BlobToolKit	4.3.7	https://github.com/blobtoolkit/blobtoolkit
BUSCO	5.4.3 and 5.5.0	https://gitlab.com/ezlab/busco
bwa-mem2	2.2.1	https://github.com/bwa-mem2/bwa-mem2
Cooler	0.8.11	https://github.com/open2c/cooler
DIAMOND	2.1.8	https://github.com/bbuchfink/diamond
fasta_windows	0.2.4	https://github.com/tolkit/fasta_windows
FastK	427104ea91c78c3b8b8b49f1a7d6bbeaa869ba1c	https://github.com/thegenemyers/FASTK
GoaT CLI	0.2.5	https://github.com/genomehubs/goat-cli
Hifiasm	0.16.1-r375	https://github.com/chhylp123/hifiasm
HiGlass	44086069ee7d4d3f6f3f0012569789ec138f42b84a a44357826c0b6753eb28de	https://github.com/higlass/higlass
MerquryFK	d00d98157618f4e8d1a9190026b19b471055b22e	https://github.com/thegenemyers/MERQURY.FK
MitoHiFi	2	https://github.com/marcelauliano/MitoHiFi
MultiQC	1.14, 1.17, and 1.18	https://github.com/MultiQC/MultiQC
NCBI Datasets	15.12.0	https://github.com/ncbi/datasets
Nextflow	23.04.0-5857	https://github.com/nextflow-io/nextflow
PretextView	0.2	https://github.com/wtsi-hpag/PretextView
purge_dups	1.2.3	https://github.com/dfguan/purge_dups
samtools	1.16.1, 1.17, and 1.18	https://github.com/samtools/samtools
sanger-tol/genomenote	1.1.1	https://github.com/sanger-tol/genomenote
sanger-tol/readmapping	1.2.1	https://github.com/sanger-tol/readmapping
Seqtk	1.3	https://github.com/lh3/seqtk
Singularity	3.9.0	https://github.com/sylabs/singularity
TreeVal	1.0.0	https://github.com/sanger-tol/treeval
YaHS	1.2a	https://github.com/c-zhou/yahs

The estimated Quality Value (QV) of the final assembly is 54.0 with *k*-mer completeness of 99.99%, and the assembly has a BUSCO v5.4.3 completeness of 93.6% (single = 92.6%, duplicated = 1.0%), using the diptera_odb10 reference set ( *n* = 3,285).

Metadata for specimens, barcode results, spectra estimates, sequencing runs, contaminants and pre-curation assembly statistics are given at https://links.tol.sanger.ac.uk/species/2881124.

## Methods

### Sample acquisition and nucleic acid extraction

A male adult *Tipula confusa* (specimen ID NHMUK014561543, ToLID idTipConf1) was collected from an urban garden in Luton, England, UK (latitude 51.89, longitude –0.38) on 2021-09-27 by aerial net. The specimen was collected and identified by Duncan Sivell (Natural History Museum) and preserved by dry freezing at –80 °C.

The workflow for high molecular weight (HMW) DNA extraction at the Wellcome Sanger Institute (WSI) Tree of Life Core Laboratory includes a sequence of core procedures: sample preparation; sample homogenisation, DNA extraction, fragmentation, and clean-up. The idTipConf1 sample was weighed and dissected on dry ice ( Jay *et al.*, 2023) and tissue from the thorax was homogenised using a PowerMasher II tissue disruptor ( Denton *et al.*, 2023a).

HMW DNA was extracted in the WSI Scientific Operations core using the Automated MagAttract v2 protocol ( Oatley *et al.*, 2023). The DNA was sheared into an average fragment size of 12–20 kb in a Megaruptor 3 system with speed setting 31 ( Bates *et al.*, 2023). Sheared DNA was purified by solid-phase reversible immobilisation ( Strickland *et al.*, 2023): in brief, the method employs a 1.8X ratio of AMPure PB beads to sample to eliminate shorter fragments and concentrate the DNA. The concentration of the sheared and purified DNA was assessed using a Nanodrop spectrophotometer and Qubit Fluorometer and Qubit dsDNA High Sensitivity Assay kit. Fragment size distribution was evaluated by running the sample on the FemtoPulse system.

Protocols developed by the WSI Tree of Life laboratory are publicly available on protocols.io ( Denton *et al.*, 2023b).

### Sequencing

Pacific Biosciences HiFi circular consensus DNA sequencing libraries were constructed according to the manufacturers’ instructions. DNA sequencing was performed by the Scientific Operations core at the WSI on a Pacific Biosciences Sequel IIe instrument. Hi-C data were also generated from head tissue of idTipConf1 using the Arima2 kit and sequenced on the Illumina NovaSeq 6000 instrument.

### Genome assembly and curation

Assembly was carried out with Hifiasm ( Cheng *et al.*, 2021) and haplotypic duplication was identified and removed with purge_dups ( Guan *et al.*, 2020). The assembly was then scaffolded with Hi-C data ( Rao *et al.*, 2014) using YaHS ( Zhou *et al.*, 2023). The assembly was checked for contamination and corrected using the TreeVal pipeline ( Pointon *et al.*, 2023). Manual curation was performed using JBrowse2 ( Diesh *et al.*, 2023), HiGlass ( Kerpedjiev *et al.*, 2018) and PretextView ( Harry, 2022). The mitochondrial genome was assembled using MitoHiFi ( Uliano-Silva *et al.*, 2023), which runs MitoFinder ( Allio *et al.*, 2020) or MITOS ( Bernt *et al.*, 2013) and uses these annotations to select the final mitochondrial contig and to ensure the general quality of the sequence.

### Evaluation of final assembly

The final assembly was post-processed and evaluated with the three Nextflow ( Di Tommaso *et al.*, 2017) DSL2 pipelines “sanger-tol/readmapping” ( Surana *et al.*, 2023a), “sanger-tol/genomenote” ( Surana *et al.*, 2023b), and “sanger-tol/blobtoolkit” ( Muffato *et al.*, 2024). The pipeline sanger-tol/readmapping aligns the Hi-C reads with bwa-mem2 ( Vasimuddin *et al.*, 2019) and combines the alignment files with SAMtools ( Danecek *et al.*, 2021). The sanger-tol/genomenote pipeline transforms the Hi-C alignments into a contact map with BEDTools ( Quinlan & Hall, 2010) and the Cooler tool suite ( Abdennur & Mirny, 2020), which is then visualised with HiGlass ( Kerpedjiev *et al.*, 2018). It also provides statistics about the assembly with the NCBI datasets ( Sayers *et al.*, 2024) report, computes *k*-mer completeness and QV consensus quality values with FastK and MerquryFK, and a completeness assessment with BUSCO ( Manni *et al.*, 2021).

The sanger-tol/blobtoolkit pipeline is a Nextflow port of the previous Snakemake Blobtoolkit pipeline ( Challis *et al.*, 2020). It aligns the PacBio reads with SAMtools and minimap2 ( Li, 2018) and generates coverage tracks for regions of fixed size. In parallel, it queries the GoaT database ( Challis *et al.*, 2023) to identify all matching BUSCO lineages to run BUSCO ( Manni *et al.*, 2021). For the three domain-level BUSCO lineage, the pipeline aligns the BUSCO genes to the Uniprot Reference Proteomes database ( Bateman *et al.*, 2023) with DIAMOND ( Buchfink *et al.*, 2021) blastp. The genome is also split into chunks according to the density of the BUSCO genes from the closest taxonomically lineage, and each chunk is aligned to the Uniprot Reference Proteomes database with DIAMOND blastx. Genome sequences that have no hit are then chunked with seqtk and aligned to the NT database with blastn ( Altschul *et al.*, 1990). All those outputs are combined with the blobtools suite into a blobdir for visualisation.

All three pipelines were developed using the nf-core tooling ( Ewels *et al.*, 2020), use MultiQC ( Ewels *et al.*, 2016), and make extensive use of the Conda package manager, the Bioconda initiative ( Grüning *et al.*, 2018), the Biocontainers infrastructure ( da Veiga Leprevost *et al.*, 2017), and the Docker ( Merkel, 2014) and Singularity ( Kurtzer *et al.*, 2017) containerisation solutions.

Table 3 contains a list of relevant software tool versions and sources.

### Wellcome Sanger Institute – Legal and Governance

The materials that have contributed to this genome note have been supplied by a Darwin Tree of Life Partner. The submission of materials by a Darwin Tree of Life Partner is subject to the **‘Darwin Tree of Life Project Sampling Code of Practice’,** which can be found in full on the Darwin Tree of Life website here. By agreeing with and signing up to the Sampling Code of Practice, the Darwin Tree of Life Partner agrees they will meet the legal and ethical requirements and standards set out within this document in respect of all samples acquired for, and supplied to, the Darwin Tree of Life Project. 

Further, the Wellcome Sanger Institute employs a process whereby due diligence is carried out proportionate to the nature of the materials themselves, and the circumstances under which they have been/are to be collected and provided for use. The purpose of this is to address and mitigate any potential legal and/or ethical implications of receipt and use of the materials as part of the research project, and to ensure that in doing so we align with best practice wherever possible. The overarching areas of consideration are:

• Ethical review of provenance and sourcing of the material

• Legality of collection, transfer and use (national and international) 

Each transfer of samples is further undertaken according to a Research Collaboration Agreement or Material Transfer Agreement entered into by the Darwin Tree of Life Partner, Genome Research Limited (operating as the Wellcome Sanger Institute), and in some circumstances other Darwin Tree of Life collaborators.

## Data Availability

European Nucleotide Archive: *Tipula confusa*. Accession number PRJEB59392; https://identifiers.org/ena.embl/PRJEB59392 ( Wellcome Sanger Institute, 2023). The genome sequence is released openly for reuse. The *Tipula confusa* genome sequencing initiative is part of the Darwin Tree of Life (DToL) project. All raw sequence data and the assembly have been deposited in INSDC databases. The genome will be annotated using available RNA-Seq data and presented through the Ensembl pipeline at the European Bioinformatics Institute. Raw data and assembly accession identifiers are reported in Table 1.

## Author information

Members of the Natural History Museum Genome Acquisition Lab are listed here: https://doi.org/10.5281/zenodo.7139035.

Members of the Darwin Tree of Life Barcoding collective are listed here: https://doi.org/10.5281/zenodo.4893703.

Members of the Wellcome Sanger Institute Tree of Life Management, Samples and Laboratory team are listed here: https://doi.org/10.5281/zenodo.10066175.

Members of Wellcome Sanger Institute Scientific Operations: Sequencing Operations are listed here: https://doi.org/10.5281/zenodo.10043364.

Members of the Wellcome Sanger Institute Tree of Life Core Informatics team are listed here: https://doi.org/10.5281/zenodo.10066637.

Members of the Tree of Life Core Informatics collective are listed here: https://doi.org/10.5281/zenodo.5013541.

Members of the Darwin Tree of Life Consortium are listed here: https://doi.org/10.5281/zenodo.4783558.
