# Supplementary material for: The genome sequence of a cranefly, Tipula ( Savtshenkia) confusa van der Wulp, 1883
Source: Wellcome Open Res. 2024 Jun 24;9:337. [Version 1] doi: 10.12688/wellcomeopenres.22470.1 (PMC11489842; doi:10.12688/wellcomeopenres.22470.1)
Supplement: Peer review history

# Reviewer report: Reviewer response for version 1

**DOI:** 10.21956/wellcomeopenres.24758.r100665

**Published:** 2024-10-18

**Authors:** Andrew Sweet

**Affiliations:** 1 Arkansas State University, Jonesboro, Arkansas, USA

This data note reports the genome sequence and a near-complete assembly of a species of cranefly. The report includes details about the nuclear chromosomes and the mitochondrial genome. The methodology and results are clear, but I have a few suggestions that could improve the report, primarily focused on adding more context to the results.

  

- Background: identifying females “may” require more care or “does” require more care?
- Please add a few more details on identifying larvae and pupae. The adults get more details, but a summary of the traits used in Brindle (1960) would be helpful.
- Instead of “on the wing,” consider “have an adult stage.”
- Does “older literature” refer to literature before 1883, or have there been synonymy issues?
- “The genome was sequenced from an adult male…”
- The BUSCO scores seem somewhat low. What are expected completeness scores for a high-quality dipteran genome?
- How does the genome size compare to other tipulids? Other dipterans?

Are sufficient details of methods and materials provided to allow replication by others?

Yes

Is the rationale for creating the dataset(s) clearly described?

Yes

Are the datasets clearly presented in a useable and accessible format?

Yes

Are the protocols appropriate and is the work technically sound?

Yes

Reviewer Expertise:

Evolutionary genomics, mitogenomics

I confirm that I have read this submission and believe that I have an appropriate level of expertise to confirm that it is of an acceptable scientific standard.

# Reviewer report: Reviewer response for version 1

**DOI:** 10.21956/wellcomeopenres.24758.r100662

**Published:** 2024-10-09

**Authors:** Jan Ševčík

**Affiliations:** 1 University of Ostrava, Ostrava, Czech Republic

The first genomic data are presented for the cranefly species *Tipula confusa*. Both chromosome and mitogenome assemblies appear as of high quality. They are already available on the GenBank database. The authors followed standard procedures of genomic sequencing within the Tree of Life project. This paper is thus a valuable contribution to the understanding the evolution of craneflies and lower Diptera as a whole.

**Minor comments:**

- Subgenus *Savtchenkia* is mentioned in the title, as well as in the text. Please briefly summaries its main diagnostic characters. Subgenera can be elevated to genus level in the future, so it is important to characterize the subgenus as well.
- The fifth paragraph mentions the name *Tipula* *marmorata*, but does not explain why this older name does not apply. If they are synonyms, the older name should be valid.
- It is not stated in the text which parts of the body were used for DNA isolation. Were the terminalia left as a voucher for further study? Were they prepared on a microscope slide or kept in a micro tube with glycerol? Would it be possible to publish a photograph of the most important diagnostic character of the species (sternite 8)?
- I would appreciate a brief comparison of the new assemblies with genomes and mitogenomes of other *Tipulidae*, or lower Diptera as a whole. Is there anything unusual in the genome or transcriptome of *Tipula obscura*? Are there any previous records of any part of its genome, e.g. COI barcode in the GenBank or BOLD databases? If so, are there any discrepancies?

Are sufficient details of methods and materials provided to allow replication by others?

Yes

Is the rationale for creating the dataset(s) clearly described?

Yes

Are the datasets clearly presented in a useable and accessible format?

Yes

Are the protocols appropriate and is the work technically sound?

Yes

Reviewer Expertise:

Taxonomy and phylogeny of lower Diptera (Nematocera), DNA barcoding, molecular phylogenetics

I confirm that I have read this submission and believe that I have an appropriate level of expertise to confirm that it is of an acceptable scientific standard.
